# Excess Mortality in Italy During the COVID-19 Pandemic: Assessing the Differences Between the First and the Second Wave, Year 2020

**DOI:** 10.3389/fpubh.2021.669209

**Published:** 2021-07-16

**Authors:** Maria Dorrucci, Giada Minelli, Stefano Boros, Valerio Manno, Sabrina Prati, Marco Battaglini, Gianni Corsetti, Xanthi Andrianou, Flavia Riccardo, Massimo Fabiani, Maria Fenicia Vescio, Matteo Spuri, Alberto Mateo Urdiales, Del Manso Martina, Graziano Onder, Patrizio Pezzotti, Antonino Bella

**Affiliations:** ^1^Department of Infectious Diseases, Istituto Superiore di Sanità, Rome, Italy; ^2^Statistical Service, Istitituto Superiore di Sanità, Rome, Italy; ^3^Division of Population Register, Demographic and Living Conditions Statistics, Italian National Institute of Statistics, Rome, Italy; ^4^Department of Cardiovascular, Endocrine-Metabolic Diseases, and Aging, Istituto Superiore di Sanità, Rome, Italy

**Keywords:** COVID-19, surveillance, mortality from all causes, excess mortality, Italy

## Abstract

COVID-19 dramatically influenced mortality worldwide, in Italy as well, the first European country to experience the Sars-Cov2 epidemic. Many countries reported a two-wave pattern of COVID-19 deaths; however, studies comparing the two waves are limited. The objective of the study was to compare all-cause excess mortality between the two waves that occurred during the year 2020 using nationwide data. All-cause excess mortalities were estimated using negative binomial models with time modeled by quadratic splines. The models were also applied to estimate all-cause excess deaths “not directly attributable to COVD-19”, i.e., without a previous COVID-19 diagnosis. During the first wave (25th February−31st May), we estimated 52,437 excess deaths (95% CI: 49,213–55,863) and 50,979 (95% CI: 50,333–51,425) during the second phase (10th October−31st December), corresponding to percentage 34.8% (95% CI: 33.8%–35.8%) in the second wave and 31.0% (95%CI: 27.2%–35.4%) in the first. During both waves, all-cause excess deaths percentages were higher in northern regions (59.1% during the first and 42.2% in the second wave), with a significant increase in the rest of Italy (from 6.7% to 27.1%) during the second wave. Males and those aged 80 or over were the most hit groups with an increase in both during the second wave. Excess deaths not directly attributable to COVID-19 decreased during the second phase with respect to the first phase, from 10.8% (95% CI: 9.5%–12.4%) to 7.7% (95% CI: 7.5%–7.9%), respectively. The percentage increase in excess deaths from all causes suggests in Italy a different impact of the SARS-CoV-2 virus during the second wave in 2020. The decrease in excess deaths not directly attributable to COVID-19 may indicate an improvement in the preparedness of the Italian health care services during this second wave, in the detection of COVID-19 diagnoses and/or clinical practice toward the other severe diseases.

## Introduction

Italy has been the first European country to experience the spread of the Sars-Cov2 virus at the end of February 2020, with the first related death occurring on February 21. A first epidemic wave was observed between the end of February and May 2020, with a peak observed in March and April (1, 2). The first wave was characterized by a great geographical heterogeneity: the northern regions were the most affected, also in terms of mortality, while in the center and the South, the epidemic had a lower impact. On the contrary, during the second wave (October–December), the virus spread was more homogeneous throughout the country (1, 2).

Following a 2-month lockdown period, the number of new cases and deaths was largely reduced during the summer period (from June to September 2020), while a second epidemic wave occurred in the country from the second half of October (3). The two epidemic waves were characterized by a substantial number of COVID-19 related deaths (4). The estimation of the excess deaths from all causes is considered the most reliable method to comprehensively evaluate the impact of COVID-19 on mortality (5). The assessment of total deaths can help to better estimate the overall impact of COVID-19, by overcoming possible issues related to underreporting of COVID-19 deaths and by assessing “indirect mortality,” i.e., caused by health systems not being able to cope with other acute or chronic conditions (6). The purpose of this work was to highlight the differences in excess deaths between the two waves that occurred in 2020.

We, then, estimated the excess mortality during the first wave by comparing it with the second in terms of geographical distribution, sex and age groups. Therefore, we also estimated the excess mortality “not directly attributable to COVID-19” to assess the possible impact of deaths without a COVID-19 diagnosis during the two waves in Italy.

## Methods, Outcomes, Data Sources, and the Choice of the Study Periods

The primary outcome variable of the study was the daily excess mortality, defined as “the difference between the observed numbers of deaths in specific time periods and the number of expected deaths in the same time periods” (7). We estimated daily expected deaths as the average of the previous 5 years (i.e., 2015–2019) and observed daily deaths were those that occurred in the year 2020 during the same periods.

A secondary outcome was the daily excess mortality “not directly attributable to COVID-19”, defined as the difference between all-cause excess deaths, as just mentioned, minus the total number of deaths after a COVID-19 diagnosis. The estimates for excess mortality were based on data provided by the Italian National Institute of Statistics (ISTAT) which, on an annual basis, gathers all-cause mortality data by day and by geographical unit by the “provinces,” which are administrative local units of Italy (8). Data on deaths with a COVID-19 diagnosis came from national COVID-19 surveillance (9). The surveillance system contains data on all laboratory-confirmed (by real-time-PCR, RT-PCR) cases of COVID-19, as already published by Riccardo et al. (10). Deaths were considered as COVID-19 related when occurring in persons who tested positive for Sars-Cov2 *via* RT-PCR and reported to the national COVID-19 surveillance (9).

To estimate the overall excess mortality during 2020, we took into consideration the period from 1st January to 31st December. Then, we divided the study period according to the waves observed in the excess mortality pattern. We defined wave or phase, as periods characterized by “a rising number of excess deaths >0 with a defined peak, followed by a decline in deaths, in which excess deaths had decreased”, as already reported for the COVID-19 epidemic (11). Therefore, we defined “transitional phase” as the time period elapsed between waves. After a preliminary graphical analysis of data [Supplementary Figure 1 by locally estimated scatterplot smoothing (LOESS) method], we divided the study period, on the basis the previous definitions, into the following phases: (i) the first wave, from 25th February 2020 to 31st May 2020; (ii) the transitional phase, from 1st June 2020 to 9th October 2020 and (iii) the second wave, from 10th October 2020 to 31st December 2020 (last date mortality data update). In this regard, we also performed a sensitivity analysis using data from national COVID-19 surveillance (9), confirming similar waves in the pattern of deaths after a COVID-19 diagnosis (Supplementary Figure 2).

## Statistical Analysis

We used statistical models to estimate both excess mortality by counts and percentage with 95% CI. In detail, we applied negative binomial models to account for over dispersion observed in the death counts distribution (i.e., to obtain a model with a chi-squared/degree of freedom more closer to 1). The daily count of deaths was the outcome variable of the employed models, whose covariates were the following: time, a categorical variable coded as 1 for the year 2020 and as 0 for the previous pre-pandemic years (2015–2019) and the interaction of the two. The time period from 1st January to 31st December was modeled by quadratic spline functions to account for seasonality. To estimate the excess mortality, we summed the exponentiated linear prediction obtained from the described model (and its 95% CIs), as reported elsewhere (12). The estimates of excess deaths with its 95% CI were obtained considering the time period of 1st January−31st December, and according to the waves observed, as well as the transitional phase, as we defined. Furthermore, we applied separate models for three macro-geographical areas (northern regions, central and southern regions), as well as for sex and age groups (0–49, 50–79 and 80+ years). The same negative binomial models were also applied for the secondary outcome, as defined previously, to give an estimate of excess mortality “not directly attributable to COVID-19” during the two epidemic waves.

Finally, given the arbitrariness of the reference period considered (i.e., 2015–2019), we made a sensitivity analysis by repeating the negative binomial models for overall all-cause excess deaths choosing as a reference each of the 5 years separately. All statistical analyses were performed using SAS 9.4.

## Results

During the year 2020 (from 1st January to 31st December), overall all-cause excess deaths in Italy were 100,526 (95% CI: 97,575–103,560) (Table 1); the corresponding percentage of excess mortality was 15.6% (95%:14.6%–16.6%). With regards to the macro-geographical area (Table 1), the majority of all-cause excess deaths were observed in northern regions of Italy: 74,295 (95% CI: 72,697–75,925) with a corresponding percentage increase of 24.6%; followed by south: 16,328 (95% CI: 15 948–16 712), with a percentage increase of 7.5% and Central Italy: 9,903 (95% CI: 9 650–10 158), with a percentage increase of 7.7%.

**Table 1:** All-cause excess deaths estimates, Italy - year 2020.

	**Deaths in** **2020**	**Expected** **deaths^**a**^**	**Excess** **deaths**	**95% CI**
Italy	746 146	645 620	100 526	(97 575–103 560)
Northern regions^b^	376 181	301 886	74 295	(72 697–75 925)
Central regions^c^	141 550	131 647	9 903	(9 650–10 158)
Southern regions^d^	228 415	212 087	16 328	(15 948–16 712)

a *The average of deaths occurred in 2015–2019;*

b *Northern regions: Piedmont, Valle d'Aosta, Liguria, Lombardy, Trentino-Alto Adige, Veneto, Friuli-Venezia Giulia, and Emilia-Romagna;*

c *Central regions: Tuscany, Umbria, Marche, Latium;*

d *Southern regions: Abruzzo, Molise, Campania, Apulia, Basilicata, Calabria, Sicily, and Sardinia*.

The results of the sensitivity analyses were reported in Supplementary Figure 3 (a, b, c, d, and e) and Supplementary Table 1: the estimates of overall all-cause excess deaths were not significantly different from that reported in Table 1 [i.e., 100,526 (95% CI: 97 575–103 560)] when chosen as reference last 2 years, i.e., 2018 or 2019. Instead, when considering as a reference the preceding years 2015, 2016, or 2017, the overall all-cause excess deaths were lower when chosen, as a reference, 2015 or 2017, whilst it was higher when we considered 2016.

### Estimates of All-Cause Excess Deaths According to the Defined Phases

**Figure 1:**
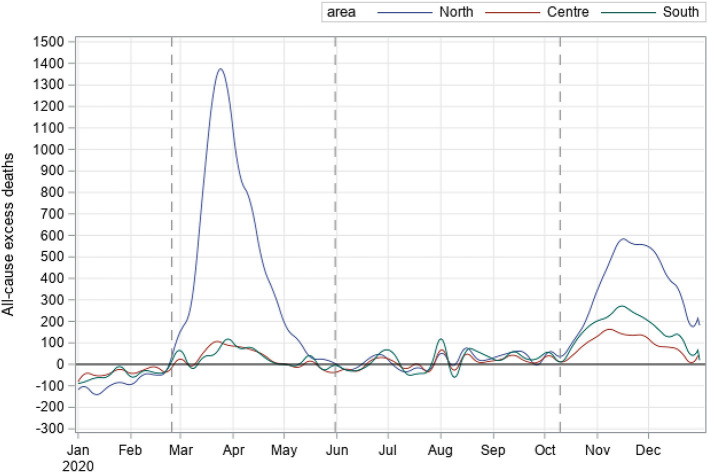
All-cause excess deaths during the COVID-19 pandemic by geographical macro-area, Italy - year 2020.

**Figure 2:**
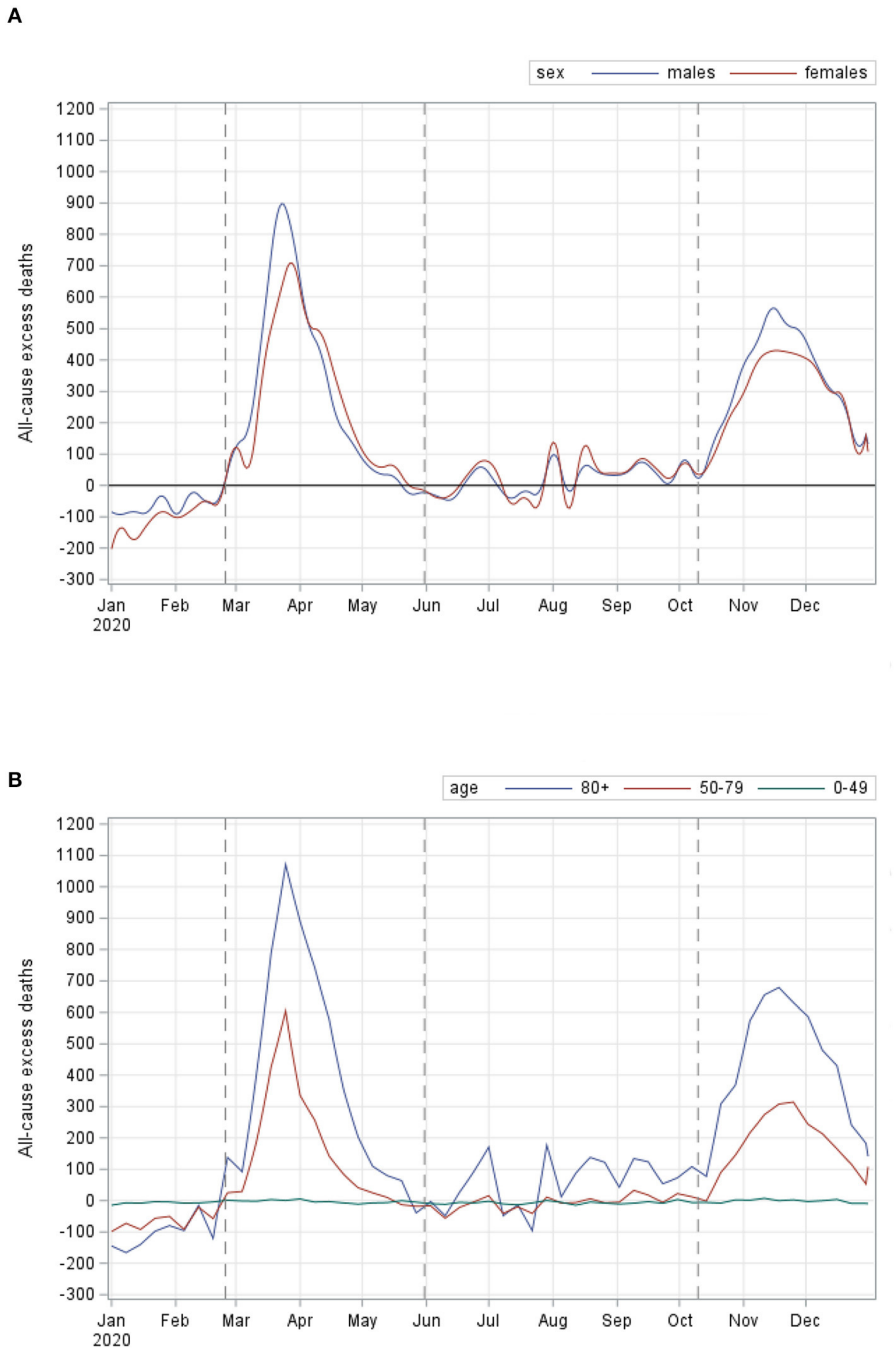
All-cause excess deaths during the COVID-19 pandemic by sex **(A)** and by age groups **(B)**, Italy - year 2020.

As described in the Methods section, we divided the overall study period (the year 2020) into three phases, mainly individuated by the waves, as clearly seen in Supplementary Figure 1. Northern Italy was the most hit macro-area in terms of excess deaths during both waves (Figure 1). We estimated counts and percentage terms for northern regions: 46,342 (95% CI: 43,922–48,891), which corresponded to 59.1% (95% CI: 52.6%–66.4%) during the first wave; whilst during the second wave, we estimated 30,023 excess deaths (95% CI: 29,675–30,371), which corresponded to 42.2% (95% CI: 41.7%–45.1%). It is important to note that excess deaths estimate increased in both, central and southern, regions in terms of counts and percentage in Central Italy, from 2,879 (95%: 2,735–3,028), equivalent to 8.3% (95% CI: 7.4%−9.4%), during the first wave to 7,691 (95%: 7,548–7,833), corresponding to 25.7% (95% CI: 24.2%–27.3%), in the second wave; in southern Italy, from 3,216 (95% CI: 3,046–3 394), corresponding to 5.7% (95% CI: 5.1–6.4%), to 13,265 (95% CI: 13,072–13,458), equivalent to 28% (95% CI: 26.7%–29.4%), during the second wave. Table 2 shows excess all-cause deaths also estimated during the 3 phases, overall and according to sex and age groups. The overall estimate of excess during the first wave was: 52,437 (95% CI: 49,213–55,863), whilst in absolute terms, we estimated a slight decrease during the second wave: 50,979 (95% CI: 50,333–51,425). Of note, when considering the excess deaths on percentage terms, we estimated an increase in excess deaths during the second wave with respect to the first, although not statistically significant: 34.8% (95% CI: 33.8%–35.8%) vs. 31% (95% CI: 27.2%–35.4%) (Table 2). Concerning sex (Figure 2A), we estimated a higher number of excess deaths in males, both on absolute and percentage terms (Table 2): 27,383 (95% CI: 25,831–29,023), excess deaths for males during the first wave, with a percentage increase of 33.8%, whilst during the second wave, we estimated 27,434 (95% CI: 27,099–27,748) excess deaths, corresponding to a percentage increase of 38.8%. Excess deaths of 25,054 (95% CI: 23,645–26,541) was estimated for females during the first wave (28% increase), whereas excess deaths of 23,545 (95% CI: 23,256–23,806) (31.1% increase) in the second wave.

**Table 2:** All-cause excess deaths estimates according to the the two waves and the transitional phase.

	**Deaths in 2020**	**Expected deaths^**a**^**	**Excess deaths**	**95% CI**	**Excess deaths %**	**95% CI**
**First wave**						
Italy	221 447	169 010	52 437	(49 213–55 863)	31.0%	(27.2%−35.4%)
Males	108 307	80 924	27 383	(25 831–29 023)	33.8%	(29.8%−38.3%)
Females	113 140	88 086	25 054	(23 645–26 541)	28.4%	(25.1%−32.2%)
Age groups						
0–49	4 735	5 018	−283	(−306; −261)	−5.6%	(−6.9%; −4.6%)
50–79	71 631	56 840	14 792	(13 998–15 623)	26.1%	(23.0%−29.4%)
80+	145 081	107 152	37 928	(35 813–40 173)	35.4%	(31.5%−39.8%)
**Transitional phase**						
Italy	170 026	166 643	3 383	(3 333–3 433)	2.0%	(1.96%−2.1%)
Males	81 267	79 868	1 399	(1 378–1 420)	1.7%	(1.6%−1.8%)
Females	88 759	86 775	1 984	(1 956–2 012)	2.3%	(2.2%−2.4%)
**Age groups**						
0–49	4 764	5 571	−807	(−851; −763)	−14.5%	(−16.9%; −7.9%)
50–79	55 722	56 834	−1 112	(−1 130; −1 092)	−2.0%	(−2.1%; −1.9%)
80+	109 540	104 238	5 302	(5 220–5 384)	5.1%	(4.9%−5.3%)
**Second wave**						
Italy	197 502	146 523	50 979	(50 533–51 425)	34.8%	(33.8–35.8%)
Males	98 204	70 770	27 434	(27 109–27 758)	38.8%	(37.3–40.3%)
Females	99 298	75 753	23 545	(23 270–23 819)	31.1%	(29.9–32.3%)
Age groups						
0–49	4 125	4 235	−110	(−118; −103)	−2.6%	(−3.1%; −2.0%)
50–79	63 983	49 070	14 913	(14 698–15 127)	30.4%	(29.0–31.8%)
80+	129 394	93 218	36 176	(35 801–36 550)	38.8%	(37.5–40.1%)

a *Average of deaths occurred in 2015–2019*.

During both the waves, the age groups with the largest numbers of excess deaths were 80 or over, as shown in part b of Figure 2. Specifically (Table 2), we estimated all-cause excess deaths of 37,929 (95% CI: 35,813–40,173) during the first wave, with a corresponding percentage increase of 35.4% and 36,176 (35,811–36,542) during the second wave, with a percentage increase of 38.8% (Table 2). Excess deaths during the first wave were 14,792 (95% CI: 13,970–15,663) in the age group 50–79, corresponding to a percentage increase of 26.1%, whilst during the second wave, corresponding to a percentage increase of 30.4%. We estimated all-cause excess deaths of −283 (95% CI: −306; −261) in age group <50 during the first wave, whilst it increased slightly [−110 (95% CI: −117; −103)] during the second wave, in terms of the percentage, this corresponded to a decrease of −5.6% during the first wave and −2.6% during the second wave. The estimates of excess deaths during the transitional phase were 3,383 (95%: 3,333–3,433), corresponding to a percentage increase of 2%. Of note, during this phase, the worst-hit age group in terms of the number of excess deaths was again 80 or over, with 5,302 deaths (95%: 5,220–5,384), corresponding to an increment of 5.1%.

### Estimates of All-Cause Excess Deaths Not Directly Attributable to COVID-19 According to the Two Defined Waves

**Figure 3:**
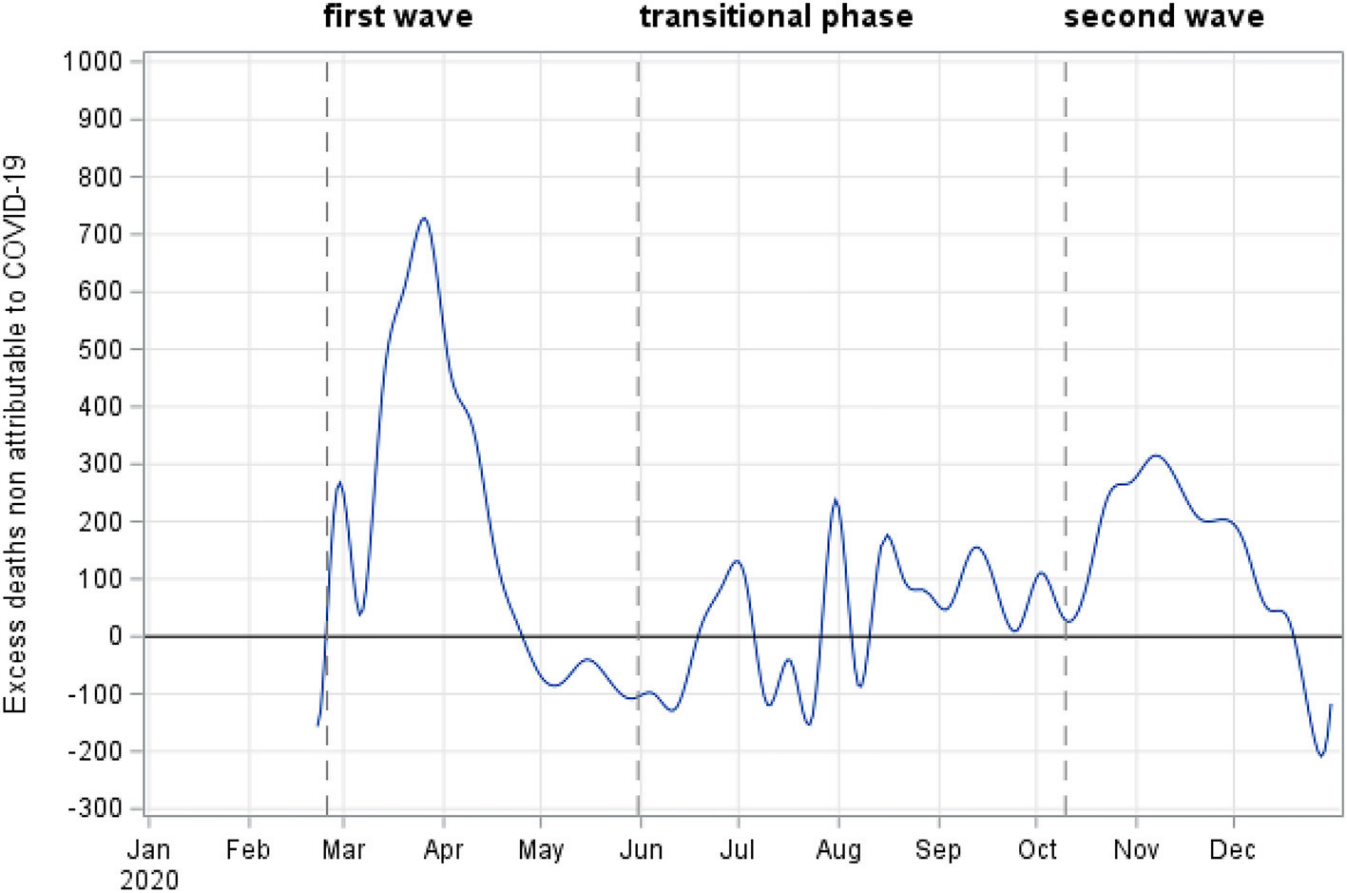
Excess deaths not directly attributable to COVID-19 during the pandemic, Italy - year 2020.

Figure 3 and Table 3 show the estimates of excess deaths that were not directly attributable to COVID-19, i.e., without a previous COVID-19 diagnosis. During the first wave, we estimated excess deaths that occurred without a previous COVID-19 diagnosis of 18,307 (95% CI: 17,197–19,487), corresponding to 10.8% (95% CI: 9.5%–12.4%). The estimate of excess deaths without a previous COVID-19 diagnosis decreased significantly during the second wave, being 11,318 (95% CI: 11,221–11,414), corresponding to 7.7% (95% CI: 7.5–7.9%). In Northern Italy (results were not showed in Table 3), we estimated percentage of excess death without a previous COVID-19 diagnosis of 22% (95% CI: 19.6%–24.8%) during the first wave, whilst the second wave, the estimate was 8.8% (95% CI: 8.5%–9.2%). On the contrary, in the rest of Italy, excess deaths without a previous COVID-19 increased from 1.1 (95% CI: 1.0–1.2%) to 6.7% (95%CI: 6.4–7.1%). A significant decrease in excess deaths not directly attributable to COVID-19 was observed in both sexes during the second wave (Table 3), as also in all age groups except for those aged <50, for whom we observed a slight increase [during the first wave: −652 (95% CI: −702; −604), during the second wave: −532, (95% CI: −563; −501)].

**Table 3:** Estimates of excess deaths “not directly attributable to COVID-19”.

	**Deaths *non*** **COVID-19^**a**^**	**Excess deaths *non*** **COVID-19^**a**^**	**95% CI**	**Excess deaths *non*** **COVID-19%^**a**^**	**95% CI**
**First wave**					
Italy	187 322	18 307	(17 197–19 487)	10.8%	(9.5–12.4%)
Males	88 520	7 593	(7 166–8 047)	9.3%	(8.3–10.6%)
Females	98 802	10 714	(10 108–11 356)	12.2%	(10.7–13.8%)
**Age groups**					
0–49	4 366	−652	(−702; −604)	−12.3%	(−15.8%; −10.6%)
50–79	57 986	1 145	(1 091–1 201)	2.0%	(1.8–2.3%)
80+	124 970	17 815	(16 863–18 821)	16.6%	(14.8–18.7%)
**Second wave**					
Italy	157 841	11 318	(11 221–11 414)	7.7%	(7.5–7.9%)
Males	76 108	5 338	(5 273–5 403)	7.5%	(7.2–7.8%)
Females	81 733	5 980	(5 909–6 051)	7.9%	(7.6–8.2%)
Age groups					
0–49	3 703	−532	(−563; −501)	−12.6%	(−14.8%; −10.6%)
50–79	50 080	1 010	(997–1 023)	2.1%	(2.0–2.2%)
80+	104 058	10 840	(10 724–10 957)	11.6%	(11.2–12.0%)

a *Deaths “not directly attributable to COVID-19”, i.e., deaths defined as “without a previous COVID-19 diagnosis”*.

## Discussion

This study provides estimates of the excess mortality, i.e., the difference between the observed number of deaths during a given time period and the expected number of deaths in the same period, occurred in Italy in 2020. The COVID-19 pandemic scenario in Italy during this period can be summarized in three phases, similarly to other European countries (5): a first wave, from late February to the end of May, characterized by a sharp increase of cases and deaths and by a high territorial concentration, in Italy mostly in the north; a transitional phase, from June to mid-September, with a low diffusion of the virus (13); and a second wave, starting from the end of September 2020, when the cases increased rapidly again until the first half of November (9) and then decreased again. We estimated an increase in all-cause excess deaths during the second wave from 31% (95% CI: 27.2%–35.4%) to 34.8% (95% CI: 33.8%–35.8%), although not statistically significant. This increase may have depended on factors including different pattern of the Sars-Cov2 diffusion observed during the second wave, both in terms of quantitative and the geographical distribution (9). In particular, a significant decrease in excess mortality in the northern regions (from 60% during the first phase to 42% in the second phase) was coupled with a significant increase in the rest of Italy. This result may be due to greater preparedness of the health care services in the north of Italy during the second wave, although the overall contribution to excess mortality during the whole year 2020 remained significantly higher in the north than in the rest of Italy.

The curve tracking all-cause excess deaths increased much faster during the first wave than in the second wave, even though the percentage of all-cause excess deaths was higher during the second wave. This result may be explained by the different mitigation measures adopted in Italy during the two phases. During the first phase, Italy was the first European country (and second only to China in the world) to adopt a hard national lockdown in March and April, whereas a different containment strategy was adopted in the autumn of 2020, based on regional parameters that resulted in a three-color classification of regions (yellow, orange, and red). Each color corresponded to a different risk scenario, from the lowest to the highest and characterized by different prevention measures against the diffusion of COVID-19 (refer to ministerial decree published in the Official Gazette, General Series No. 275, 4th November 2020, ordinary supplement No. 41).

Focusing on gender differences, as widely reported in the literature (14) and despite a similar incidence of COVID-19 cases reported for men and women (9), men presented a higher mortality risk than women (15). This difference appeared to be more marked during the second wave, even though not significantly.

Not surprisingly, the highest contribution to excess mortality during the whole period covered by this study was observed among people aged 80 years and older, with a further increase during the second wave (from 35% in the first to 39% in the second wave). It is important to note that this age group is particularly relevant in Italy, where it accounts for 7.4% of the population, compared with an EU average of 5.9%.

Another core finding of this study is that the excess mortality observed among people aged less than 50, although of negative sign, increased slightly during the second wave. The most likely explanation is that the COVID-19 related deaths recorded in this part of the population concern, in most cases, people who were already suffering from serious diseases, that is, a very fragile component of the population (16).

During the second wave, we also estimate a decrease in the excess deaths that are not directly attributable to COVID-19 (i.e., without a previous COVID-19 diagnosis), which we approximated by using a proxy. This finding can be interpreted as an improvement recorded by the national health system in the diagnosis and treatment of other severe diseases that were previously delayed by the presence of COVID-19, which was overburdening the health care services during the first wave. As a result, there was a decline in the indirect effects of the COVID-19 pandemic on other diseases during the second wave when compared with the first one.

Another explanation for the decrease in excess deaths not directly attributable to COVID-19 could be the improvement in the detection of COVID-19 related cases *via* diagnoses completed before death. To support this aim, the National Institute of Statistics has recently reported data on specific causes of deaths (see https://www.istat.it/it/archivio/256854), which showed that COVID-19 was the first cause of death in Italy during the period March–April 2020: overall, 60% of deaths were attributable to COVID-19, 10% to pneumonia, most likely related to COVID-19 and 30% to other causes. While findings are yet to be published for the second wave, the decline of excess deaths not directly attributable to COVID-19 estimated in this study also seems to confirm the higher proportion of deaths attributable to COVID-19 in this second phase.

We are aware that a limitation in our study may be given by the choice of the reference period (2015–2019) to estimate the excess deaths, which is rather arbitrary. Different reference periods may produce different results. We hypothesize, however, that the period chosen may provide a good benchmark as it includes both years characterized by a high mortality rate, such as 2015, and years as 2016 or 2018 characterized in Italy by lower mortality. This resulted in a lower or higher excess estimate over the years as confirmed by the supplementary analysis. In addition, as the number of deaths in a given year depends on both the multiple factors that affect survival and the age structure of the population (13), it is appropriate to choose a reference period that is as close in time as possible to the observed periods.

The lack of data on specific causes of death was a second limitation of the study. Thus, to estimate the impact of the other deaths “not directly attributable to COVID-19” we used a proxy, defined as the difference between all-cause excess deaths minus the total number of deaths after a COVID-19 diagnosis.

Furthermore, we did not consider ambient temperature that is also an important factor that can cause excess mortality (17) and the two waves occurred in two seasons with different temperature patterns. Although we applied the quadratic spline function of time to account for seasonality, there might still exist residual confounding caused by temperature.

Overall, while it is true that only a specific analysis of the actual causes of each death would allow for a fully-fledged understanding of the differences between age groups, we hypothesize that all-cause excess mortality is an effective epidemiological tool that includes both the direct and indirect effects of the COVID-19 disease.

An important strength of this study is that it was conducted by using high-quality national data provided by the ISTAT, which are exhaustive with regards to total mortality and the best available with regard to the COVID-19 cases and related deaths, as detected by the national surveillance system of the National Institute of Health (ISS). This reliable dataset has enabled to identify some findings that may contribute to a better understanding of the COVID-19 pandemic in Italy. Finally, this study includes suggestions for future research avenues that may further help in the identification of more effective public health measures.

## Data Availability Statement

The original contributions presented in the study are included in the article/Supplementary Material, further inquiries can be directed to the corresponding authors.

## Ethics Statement

Ethical review and approval was not required for the study on human participants in accordance with the local legislation and institutional requirements. Written informed consent for participation was not provided by the participants' legal guardians/next of kin because COVID-19 surveillance data are collected in the National Institute of Health in Italy (ISS) and in February 27th, 2020, the Italian Presidency of the Council of Ministers, being compliant with the European General Data Protection Regulation (UE GDPR 2016/679) authorized the processing of personal data related to COVID-19 by the ISS and other public institutions (such as ISTAT too) for reason of public interest in the area of public health. Further, data are already anonymised.

## Consent to Participate

COVID-19 surveillance data are collected in the ISS in Italy and on 27th February 2020, the Italian Presidency of the Council of Ministers, being compliant with the European General Data Protection Regulation (UE GDPR 2016/679) authorized the processing of personal data related to COVID-19 by the ISS and other public institutions, such as ISTAT, for the reason of public interest in the area of public health.

## Consent for Publication

All authors approved the final manuscript as submitted and agreed to be accountable for all aspects of the work.

## Author Contributions

MD performed the statistical analyses and drafted the manuscript. GM contributed to the design of the study and revised the advanced draft of the manuscript. SB elaborated surveillance data and revised critically the manuscript. VM elaborated mortality data. SP coordinated and supervised national mortality data. MB and GC elaborated national mortality data and revised critically the manuscript. XA, FR, MF, and MV elaborated surveillance data and revised critically the manuscript. MS, AM-U, and MDM elaborated surveillance data. GO revised critically the manuscript. PP is the head of the Italian coronavirus disease surveillance system and revised the manuscript. AB coordinated and supervised the surveillance data collection and contributed to the conception and design of the study by revising critically the manuscript. All authors contributed to the article and approved the submitted version.

## Conflict of Interest

The authors declare that the research was conducted in the absence of any commercial or financial relationships that could be construed as a potential conflict of interest.
