# Supplementary material for: Excess Mortality in Italy During the COVID-19 Pandemic: Assessing the Differences Between the First and the Second Wave, Year 2020
Source: Front Public Health. 2021 Jul 16;9:669209. doi: 10.3389/fpubh.2021.669209 (PMC8322580; doi:10.3389/fpubh.2021.669209)
Supplement: Supplementary file 1 [file Data_Sheet_1.PDF]

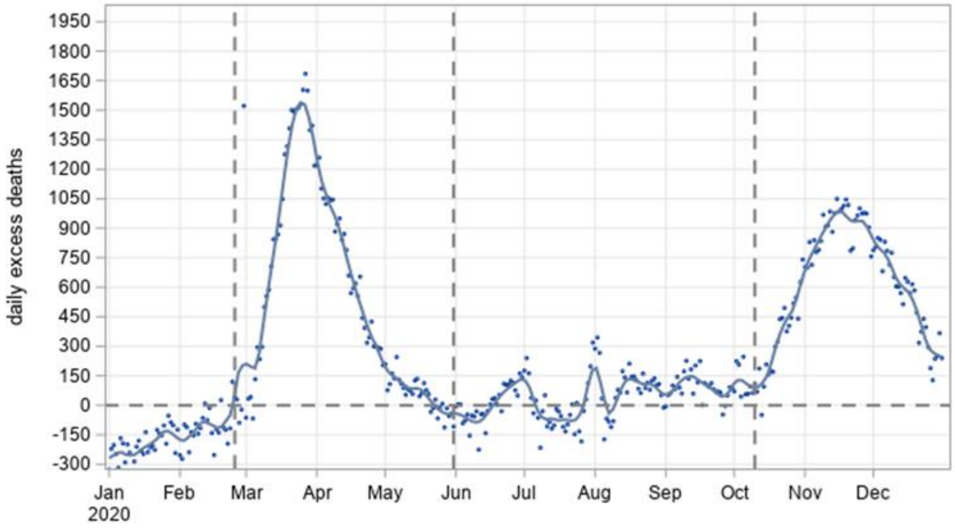

Figure 1. All-cause excess mortality in year 2020 - Italy

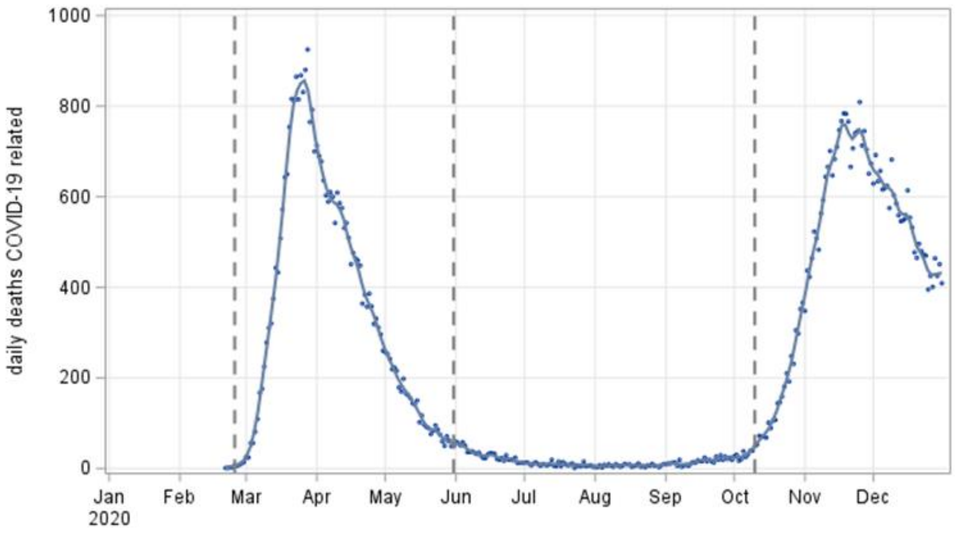

Figure 2. Deaths after COVID-19 diagnosis in year 2020 - Italy
