# Supplementary material for: Excess Mortality in Italy During the COVID-19 Pandemic: Assessing the Differences Between the First and the Second Wave, Year 2020
Source: Front Public Health. 2021 Jul 16;9:669209. doi: 10.3389/fpubh.2021.669209 (PMC8322580; doi:10.3389/fpubh.2021.669209)
Supplement: Supplementary file 2 [file Data_Sheet_2.PDF]

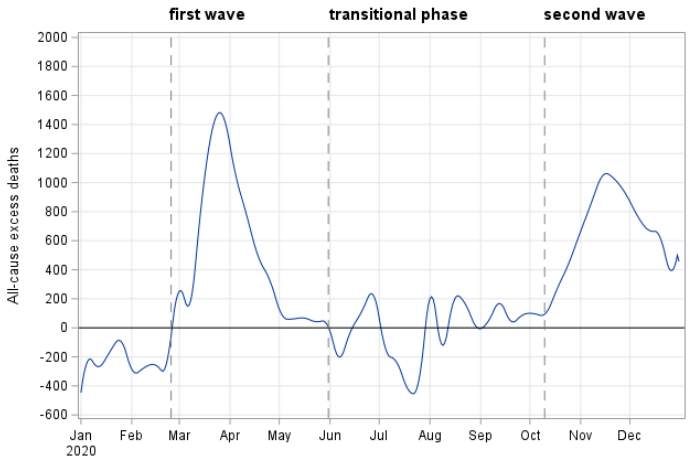

Fig.3 a) Sensitivity analysis: binomial negative model, year 2020 - year 2015.

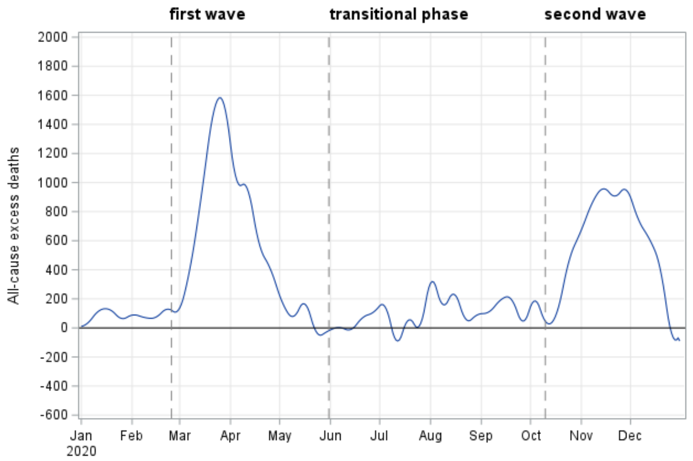

Fig.3 b) Sensitivity analysis: binomial negative model, year 2020 - year 2016.

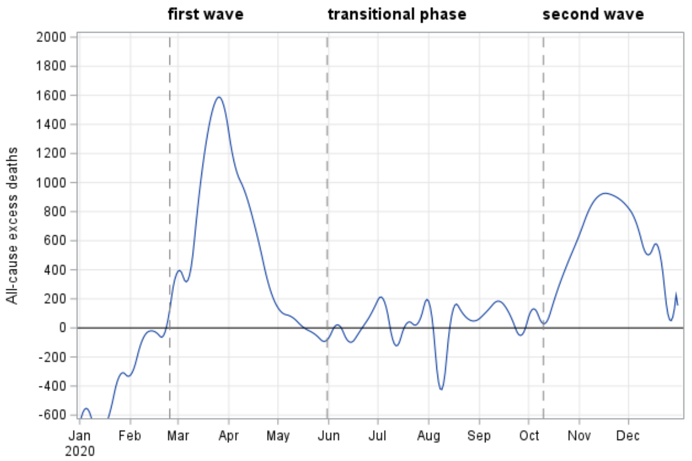

Fig.3 c) Sensitivity analysis: binomial negative model, year 2020 - year 2017.

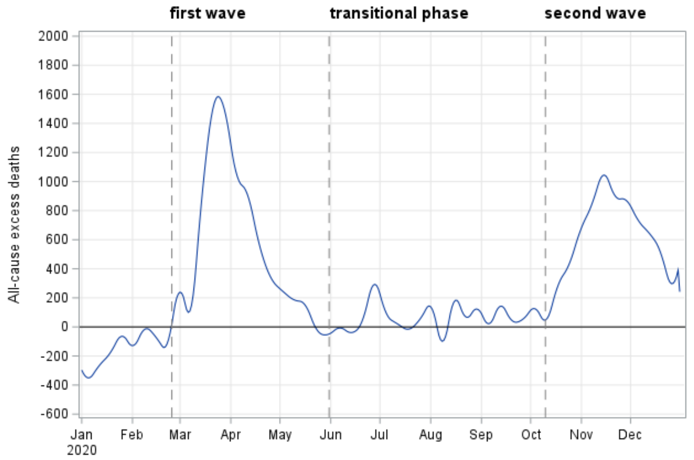

Fig.3 d) Sensitivity analysis: binomial negative model, year 2020 - year 2018.

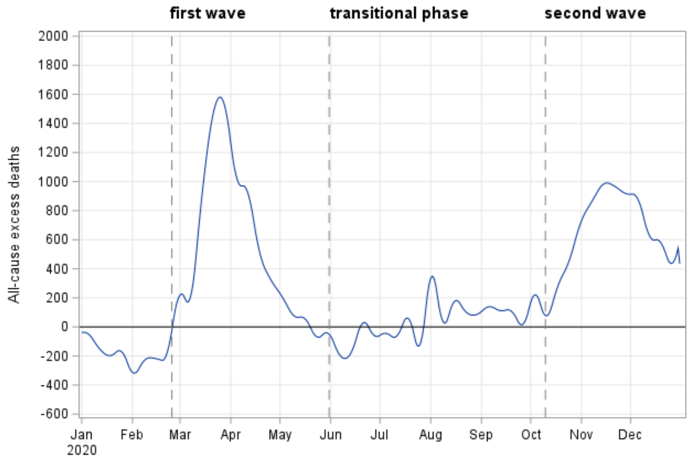

Fig.3 e) Sensitivity analysis: binomial negative model, year 2020 - year 2019.

Table *S*1. Excess deaths choosing as a reference each of the five years separately.

Ref. year	Excess deaths	95% CI	Excess deaths %	95% CI
**a) 2015**	89,950	85,143-95,025	13.7%	12.2%-15.3%
**b) 2016**	119,075	117,095-121,082	19.0%	18.2%-19.8%
**c) 2017**	86,673	82,260-91,312	13.1%	11.8%-14.6%
**d) 2018**	105,303	99,733- 111,171	16.4%	14.7%-18.4%
**e) 2019**	101,631	96,166-107,404	15.8%	14.1%-17.7%
